# Effect of the inactivation of lactate dehydrogenase, ethanol dehydrogenase, and phosphotransacetylase on 2,3-butanediol production in *Klebsiella pneumoniae* strain

**DOI:** 10.1186/1754-6834-7-44

**Published:** 2014-03-26

**Authors:** Xuewu Guo, Chunhong Cao, Yazhou Wang, Chaoqun Li, Mingyue Wu, Yefu Chen, Cuiying Zhang, Huadong Pei, Dongguang Xiao

**Affiliations:** 1 Key Laboratory of Industrial Fermentation Microbiology, Ministry of Education; Tianjin Industrial Microbiology Key Lab, College of Biotechnology, Tianjin University of Science and Technology, Box 08, No. 29, 13ST. TEDA, Tianjin 300457, China

**Keywords:** 2,3-butanediol, lactate dehydrogenase, ethanol dehydrogenase, phosphotransacetylase, *Klebsiella pneumoniae*

## Abstract

**Background:**

2,3-Butanediol (2,3-BD) is a high-value chemical usually produced petrochemically but which can also be synthesized by some bacteria. To date, *Klebsiella pneumoniae* is the most powerful 2,3-BD producer which can utilize a wide range of substrates. However, many by-products are also produced by *K. pneumoniae*, such as ethanol, lactate, and acetate, which negatively regulate the 2,3-BD yield and increase the costs of downstream separation and purification.

**Results:**

In this study, we constructed *K. pneumoniae* mutants with lactate dehydrogenase (LDH), acetaldehyde dehydrogenase (ADH), and phosphotransacetylase (PTA) deletion individually by suicide vector conjugation.

These mutants showed different behavior of production formation. Knock out of *ldhA* had little influence on the yield of 2,3-BD, whereas knock out of *adhE* or *pta* significantly improved the formation of 2,3-BD. The accumulation of the intermediate of 2,3-BD biosynthesis, acetoin, was decreased in all the mutants. The mutants were then tested in five different carbon sources and increased 2,3-BD was observed. Also a double mutant strain with deletion of *adhE* and *ldhA* was constructed which resulted in accelerated fermentation and higher 2,3-BD production. In fed-batch culture this strain achieved more than 100 g/L 2,3-BD from glucose with a relatively high yield of 0.49 g/g.

**Conclusion:**

2,3-BD production was dramatically improved with the inactivation of *adhE* and *pta.* The inactivation of *ldhA* could advance faster cell growth and shorter fermentation time. The double mutant strain with deletion of *adhE* and *ldhA* resulted in accelerated fermentation and higher 2,3-BD production. These results provide new insights for industrial production of 2,3-BD by *K. pneumoniae*.

## Background

Biorefineries for chemicals have attracted a great deal of interest because they can alleviate the dependence on oil supply for the production of platform chemicals, reduce environmental pollution, and ensure sustainable development [1,2]. Biological production of 2,3-butanediol (2,3-BD) has increased remarkably because of its wide industrial applications [3-5]. Many microorganisms classified in the genera *Enterobacter*, *Klebsiella*, *Serratia*, and *Bacillus* can produce 2,3-BD [3,4]*. K. pneumoniae* is a potentially useful producer in the industry because of its wide substrate spectrum, high efficiency, and cultural adaptability [6-8].

The fermentation pathways of mixed acid-2,3-BD in bacteria have been intensively studied [4]. Numerous studies have investigated the use of metabolic engineering to modify the metabolic pathways for improving 2,3-BD production in *Enterobacter aerogenes*[9]*, Bacillus licheniformis*[10], and *K. oxytoca*[11]. The formation of ethanol, lactate, and acetate competes with the biosynthesis of 2,3-BD for pyruvate, resulting in reduced 2,3-BD production [4,12-14]. For example, the inactivation of acetaldehyde dehydrogenase gene significantly increases the 2,3-BD yield and decreases ethanol production [11]. Deletion of the *ldh* gene encodes lactate dehydrogenase (LDH) in *E. aerogenes*, resulting in a very small amount of lactate yield and 16.7% more 2,3-BD than that of the parent strain in batch fermentation [9]. The *ldh*, *ldhB*, and *ldhX* genes in *Lactococcus lactis*, which encode LDH, have been deleted, and the results showed that *ΔldhB* is a valuable basis for engineering strategies for the production of reduced compounds [15]. The deletion of *adhE* in *K. oxytoca* increases the hydrogen yield of the mutant by 16.07% but decreases the ethanol concentration by 77.47%, compared with those of the parent strain [16].

However, only a few studies have focused on *K. pneumoniae* engineering [17-19]*.* Ji *et al*. reported that the overexpression of NOX and nox-2, which can decrease the intracellular nicotinamide adenine dinucleotide plus hydrogen (NADH)/nicotinamide adenine dinucleotide (NAD) ^+^ ratio in *K. pneumoniae*, could improve acetoin production [17]. Zhang *et al*. investigated the effect of inactivating aldehyde dehydrogenase in *K. pneumoniae* on 1,3-propanediol production [18]. However, metabolic engineering focused on 2,3-BD production in *K. pneumoniae* has not yet been intensively investigated.

*K. pneumoniae* produces 2,3-BD from a wide range of substrates but generates numerous byproducts [20], thereby increasing the cost of product downstream separation [21]. The genetic alteration of *K. pneumoniae* perhaps makes it more efficiency in industry. In this study, *K. pneumoniae* mutants with deletion of LDH, ADH and phosphotransacetylase (PTA) were constructed to evaluate the effect on 2,3-BD production. The metabolic flux and production of byproducts, such as ethanol, acetoin, lactic acid, and acetate, were also investigated.

## Results

### LDH, ADH, and PTA activity assays

**Figure 1:**
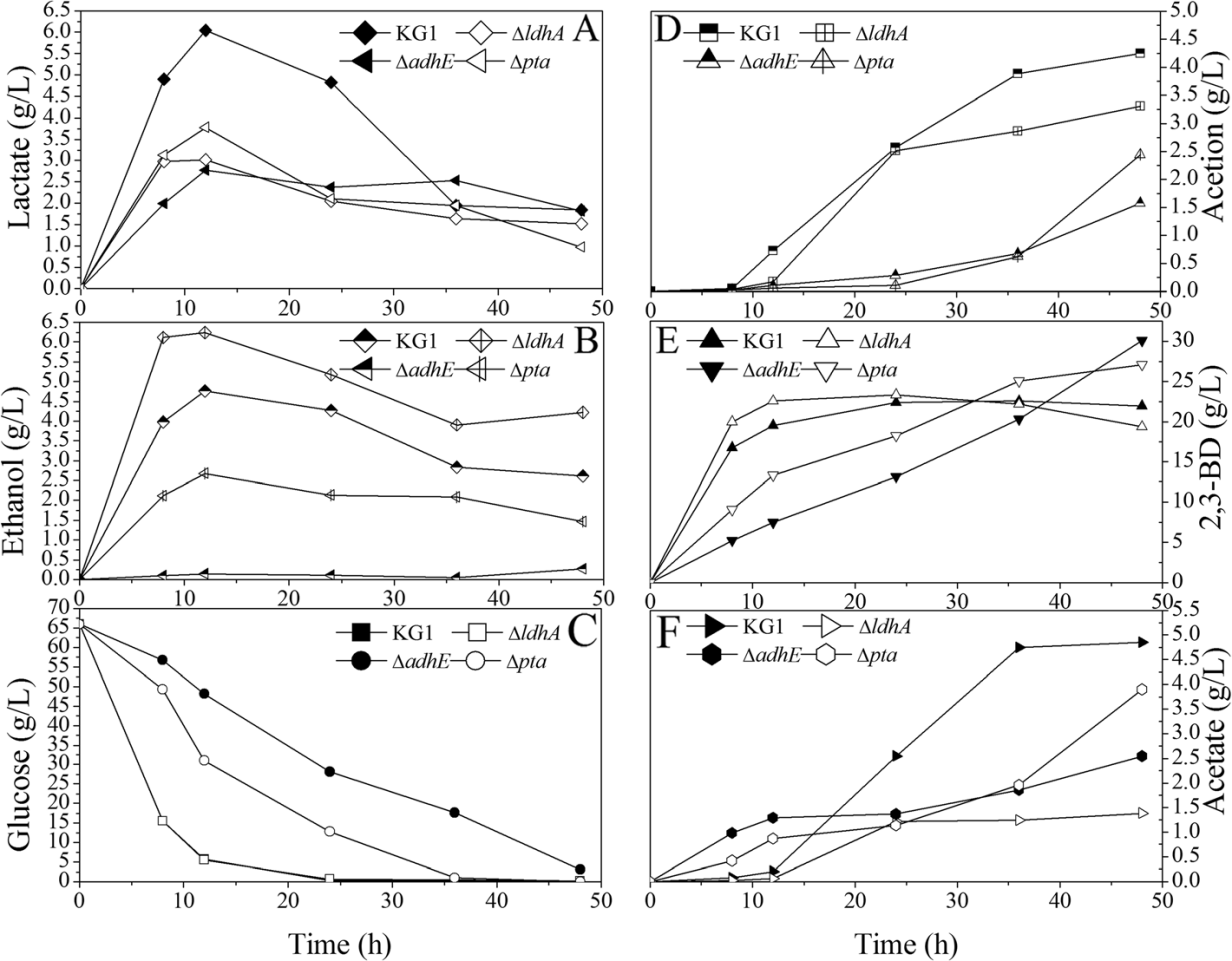
**Metabolite profiles of the *K. pneumoniae *parent strain (KG1) and the mutants in the batch culture.** KG1: the parent strain; Δ*ldhA*, Δ*adhE*, and Δ*pta*: the mutants. **A)** Lactate; **B)** Ethanol; **C)** Glucose; **D)** Acetion; **E)** 2,3-BD; **F)** Acetate. The curves were calculated from one measurement of three experiments.

The activities of LDH, ADH, and PTA in the mutants and parent strain were determined. As shown in Table 1, the LDH activities of *ldhA*, *adhE*, and *pta* deletion strains were 12.02%, 93.35%, and 97.80% of the parent strain, respectively. The PTA activities of *ldhA*, *adhE*, and *pta* deletion strains were 69.83%, 71.48%, and 1.65% of the parent strain, respectively. The ADH activities of *ldhA*, *adhE*, and *pta* deletion strains were 102.99%, 2.24%, and 85.76% of the parent strain, respectively. These results further confirmed the knockdown efficiency of these three genes respectively. Table 1 also shows that knockdown of one gene could affect the other enzyme expression or activity. For example, the Δ*ldhA* mutant showed high production of ethanol (Figure 1B), which may be caused by the high ADH activity. The production of lactate, ethanol and acetate was different in *ldhA*, *adhE*, and *pta* deletion mutants, which also proved this point. Perhaps the different metabolic pathways are complicated, and could crosstalk with each other.

**Table 1:** **Lactate dehydrogenase (LDH), phosphotransacetylase (PTA) and acetaldehyde dehydrogenase (ADH) activity of *K. pneumoniae *** **parent strain (KG1) and its mutants, the values represent mean ± SD**

**Strains**	**LDH activity**		**PTA activity**		**ADH activity**	
	**U/mg protein**	**Percentage of wild type**	**U/mg protein**	**Percentage of wild type**	**U/mg protein**	**Percentage of wild type**
KG1	4.66 ± 0.23	100	2.42 ± 0.04	100	2.67 ± 0.22	100
Δ*ldhA*	0.56 ± 0.03	12.02	1.69 ± 0.05	69.83	2.75 ± 0.21	102.99
Δ*adhE*	4.35 ± 0.31	93.35	1.73 ± 0.03	71.48	0.06 ± 0.01	2.24
Δ*pta*	4.56 ± 0.25	97.80	0.04 ± 0.01	1. 65	2.29 ± 0.15	85.76

### Effect of *ldhA*, *adhE*, and *pta* deletion on cell growth

**Figure 2:**
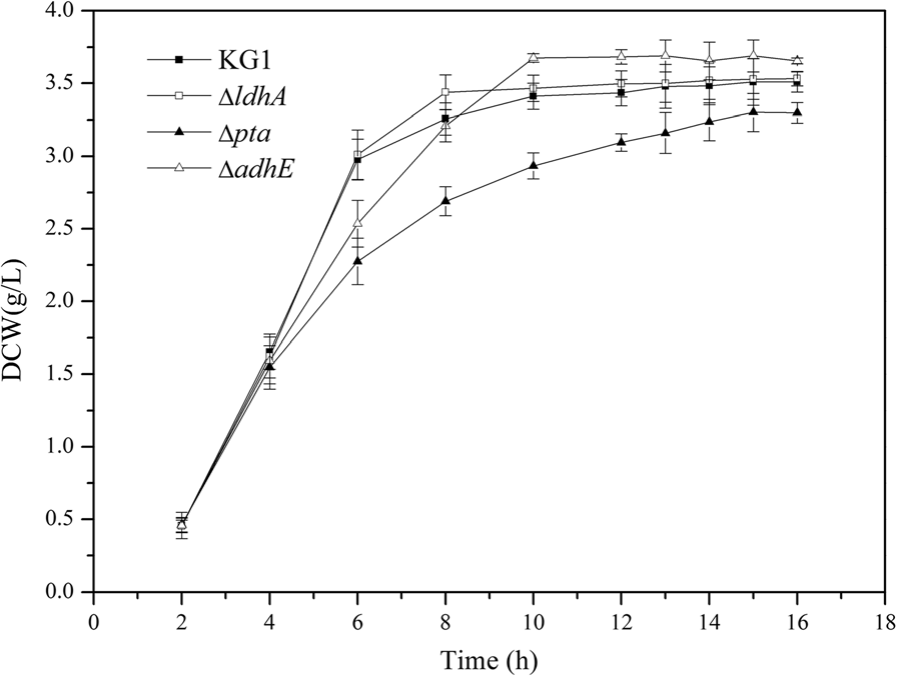
**Growth curves of the parent and mutants of *K. pneumoniae *with 20 g/L glucose.** KG1: the parent strain; Δ*ldhA*, Δ*adhE*, and Δ*pta*: the mutants. DCW, dry cell weight. The curves were calculated from one measurement of three experiments.

To investigate the effect of *ldhA*, *adhE*, and *pta* deletion on cell growth, the mutants and parent strain were cultured under the same conditions. In the first 8 h, knockout of *ldhA* promoted cell growth, whereas knockout of *adhE* and *pta* inhibited cell growth (Figure 2). The biomass accumulations of Δ*ldhA* and Δ*pta* strains were higher than that of the parent strain at 12 h. The Δ*pta* strain achieved the highest biomass compared with other mutants and parent strains at 12 h, indicating that the strain exhibited good fermentation performance, although the growth rate was lower in the first 8 h.

### Internal redox state of *ldhA*, *adhE*, and *pta* deletion strains

**Figure 3:**
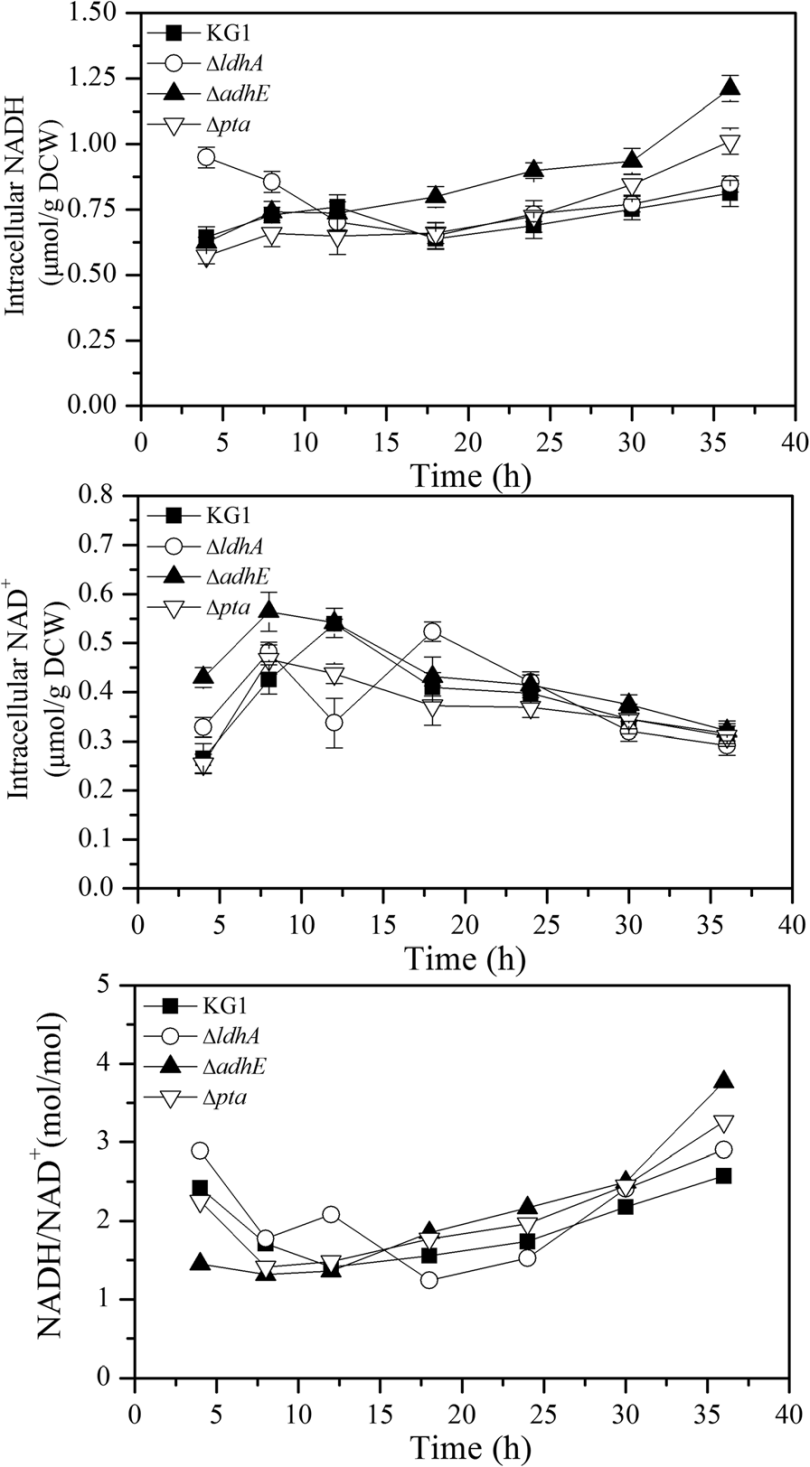
**Time courses profile of nucleotide pools in *K. pneumoniae *parent strain (KG1) and the mutants in the batch culture.** KG1: the parent strain; Δ*ldhA*, Δ*adhE*, and Δ*pta*: the mutants. The curves were calculated from one measurement of three experiments. NAD, nicotinamide adenine dinucleotide; NADH, nicotinamide adenine dinucleotide plus hydrogen; DCW, dry cell weight.

The internal redox state was investigated from 4 h to 36 h in the fed-batch fermentation. As shown in Figure 3, in *ΔldhA* mutant fermentations, the total dinucleotide pool and NADH/NAD ^+^ ratio was very high in the early exponential period, then gradually decreased in the late stationary phase, whereas, the total dinucleotide pool and NADH/NAD ^+^ ratio in *adhE* and *pta* mutants was low at 4 h to 12 h, then increased significantly from 18 h, respectively. Compared with *K. pneumoniae* parent strain (KG1), the NAD^+^ level decreased whereas NADH increased in the fermentation of *ΔadhE* and *Δpta* mutants. Thus, the NADH/NAD ^+^ ratio was much higher in the *ΔadhE* and *Δpta* from 12 h. This variation coincides with 2,3-BD flux distribution.

### Metabolic profiles of the mutants and their effect on 2,3-BD production

The concentrations of major metabolites in mutants and parent strains were investigated (Figure 1). As shown in Figure 1, three mutants showed different metabolic performance. The inactivation of *ldhA* resulted in 90% reduction in lactate production, which confirmed that *ldhA* was the major LDH manufacturing gene in *K. pneumoniae.* The 2,3-BD production of Δ*ldhA* reached 22.52 g/L at 12 h, whereas that of the parent strain was only 19.51 g/L. The maximum 2,3-BD production of Δ*ldhA* was 23.32 g/L at 24 h, whereas that of the parent strain reached maximum (22.57 g/L) at 36 h. The fermentation time was shortened from 36 h to 24 h. For byproducts, the Δ*ldhA* mutant strain produced 18.87 g/L succinate and 12.31 g/L ethanol, which were 67.4% and 53.7% higher than those in the wild-type strain. However, 2,3-BD production did not increase significantly.

The highest production of 2,3-BD was achieved at 48 h by the Δ*adhE* strain, which reached 30.10 g/L. And the highest production of 2,3-BD by the parent strain was 22.57 g/L in 36 h. Although the fermentation time was longer than the wild-type strain, 2,3-BD production increased by 33.65%, and yields of approximately 94% were obtained. The recombinant strains produced less acetoin, acetic acid, ethanol, and lactic acid, because the corresponding enzyme activity was lower..

The 2,3-BD production also reached 27.32 g/L at 48 h in the Δ*pta* strain. Lactate production was lower than the Δ*adhE* and parent strains. The Δ*pta* strain produced more acetate, acetoin, and ethanol than the Δ*adhE* strain, but their production rates were lower than those of the wild-type strain. Acetate production decreased by only 19.58% compared to that of the parent strain.

### Carbon source effects on the Δ*ldhA*, Δ*adhE*, and Δ*pta* mutants

*K. pneumoniae* can naturally use a wide spectrum of carbon sources effectively [5]. Five carbon sources, such as glucose, galactose, fructose, maltose, and lactose, were chosen to test the metabolic effects of mutants (Tables 2 and 3). As shown in Table 2, ethanol production and glucose consumption significantly increased, whereas lactate and acetate production decreased by the Δ*ldhA* strain in a flask culture with glucose, galactose, fructose, or maltose, respectively. However, 2,3-BD production slightly increased. This result suggests that the reduced pH in the *ldhA* mutant cultivation media increases 2,3-BD production [22]. Lactate and acetate production decreased, which alleviated the inhibition of cell growth. The Δ*ldhA* mutant underwent less pH shock than that of the wild-type strain; thus, a shorter fermentation time was observed.

**Table 2:** **Comparison of *K. pneumoniae *** **parent strain (KG1) and the Δ** ***ldhA * using several carbon sources in a 12-h flask cultivation, the values represent mean ± SD**

**Strain**	**Carbon source (50 g/L)**	**Consumed carbon source (g/L)**	**2,3-Butanediol (g/L)**	**L-Lactate (g/L)**	**D-Lactate (g/L)**	**Acetate (g/L)**	**Acetoin (g/L)**	**Ethanol (g/L)**	**Biomass (g/L)**	**2,3-Butanediol yield (g/g)**	**R**_**H**_	**R**_**C**_
KG1	Glucose	44.41 ± 0.45	13.82 ± 0.05	2.05 ± 0.03	3.34 ± 0.15	0.70 ± 0.04	0.65 ± 0.02	3.24 ± 0.21	4.63 ± 0.14	0.31	0.82	0.98
	Lactose	24.04 ± 0.23	5.16 ± 0.03	2.05 ± 0.02	0.53 ± 0.03	1.35 ± 0.10	0.35 ± 0.03	1.29 ± 0.05	2.42 ± 0.08	0.21	0.66	1.57
	Fructose	38.11 ± 0.32	13.26 ± 0.11	2.64 ± 0.04	1.53 ± 0.06	0.81 ± 0.06	0.54 ± 0.06	3.68 ± 0.12	4.02 ± 0.07	0.35	0.97	1.13
	Galactose	31.52 ± 0.28	10.45 ± 0.08	1.92 ± 0.02	3.31 ± 0.18	0.80 ± 0.07	0.67 ± 0.04	3.30 ± 0.11	3.49 ± 0.11	0.33	1.01	1.28
	Maltose	41.63 ± 0.35	15.05 ± 0.10	3.19 ± 0.03	2.58 ± 0.21	0.38 ± 0.02	—	4.53 ± 0.23	3.54 ± 0.05	0.36	1.24	1.30
Δ*ldhA*	Glucose	44.74 ± 0.21	15.87 ± 0.08	2.38 ± 0.03	0.05 ± 0.01	0.83 ± 0.04	1.75 ± 0.10	5.42 ± 0.32	4.84 ± 0.05	0.35	1.04	1.13
	Lactose	18.24 ± 0.15	2.97 ± 0.05	1.66 ± 0.02	0.44 ± 0.05	2.16 ± 0.04	0.19 ± 0.03	1.29 ± 0.16	1.57 ± 0.03	0.16	0.54	1.34
	Fructose	49.52 ± 0.22	16.97 ± 0.24	3.04 ± 0.04	0.21 ± 0.06	0.30 ± 0.05	0.67 ± 0.02	4.92 ± 0.18	4.49 ± 0.08	0.34	1.11	0.99
	Galactose	47.04 ± 0.28	14.33 ± 0.15	2.82 ± 0.02	0.12 ± 0.02	0.81 ± 0.03	2.11 ± 0.11	5.55 ± 0.22	4.49 ± 0.04	0.30	1.13	1.09
	Maltose	49.59 ± 0.35	18.75 ± 0.11	3.95 ± 0.07	0.08 ± 0.02	0.27 ± 0.05	1.37 ± 0.05	6.01 ± 0.24	5.06 ± 0.15	0.38	1.18	1.22

**Table 3:** **Comparison of *K. pneumoniae *** **parent strain (KG1), Δ** ***adhE * and the Δ** ***pta *** **using several carbon sources in a 36-h flask cultivation, the values represent mean ± SD**

**Strain**	**Carbon source (50 g/L)**	**Consumed carbon source (g/L)**	**2,3-Butanediol (g/L)**	**Lactate (g/L)**	**Acetate (g/L)**	**Acetoin (g/L)**	**Ethanol (g/L)**	**Biomass (g/L)**	**2,3-Butanediol yield (g/g)**
KG1	Glucose	49.29 ± 0.65	16.76 ± 0.22	3.62 ± 0.21	4.52 ± 0.45	1.73 ± 0.09	2.67 ± 0.16	4.80 ± 0.32	0.34
	Lactose	49.39 ± 0.59	16.28 ± 0.13	2.95 ± 0.15	2.98 ± 0.28	3.26 ± 0.11	4.16 ± 0.32	2.80 ± 0.43	0.33
	Fructose	44.84 ± 0.61	14.41 ± 0.16	2.27 ± 0.12	2.85 ± 0.33	2.46 ± 0.13	3.22 ± 0.25	4.06 ± 0.52	0.32
	Galactose	34.89 ± 0.48	10.27 ± 0.11	3.33 ± 0.15	2.83 ± 0.32	1.80 ± 0.10	2.64 ± 0.28	3.89 ± 0.18	0.29
	Maltose	49.20 ± 0.51	17.30 ± 0.21	2.03 ± 0.14	2.79 ± 0.16	3.56 ± 0.12	4.45 ± 0.45	3.87 ± 0.21	0.35
Δ*adhE*	Glucose	49.27 ± 0.42	23.65 ± 0.25	1.35 ± 0.09	2.05 ± 0.12	0.60 ± 0.04	0.52 ± 0.04	3.95 ± 0.52	0.48
	Lactose	17.76 ± 0.25	8.76 ± 0.12	1.61 ± 0.11	3.85 ± 0.42	2.01 ± 0.08	0.36 ± 0.06	1.76 ± 0.35	0.49
	Fructose	49.47 ± 0.52	21.40 ± 0.24	1.48 ± 0.13	1.58 ± 0.12	0.83 ± 0.06	0.94 ± 0.02	3.25 ± 0.28	0.43
	Galactose	36.35 ± 0.42	14.05 ± 0.42	0.73 ± 0.07	1.96 ± 0.09	3.70 ± 0.21	0.65 ± 0.03	2.99 ± 0.42	0.39
	Maltose	49.49 ± 0.47	18.18 ± 0.23	2.96 ± 0.10	1.27 ± 0.08	4.58 ± 0.24	0.91 ± 0.08	4.27 ± 0.25	0.36
Δ*pta*	Glucose	49.27 ± 0.54	20.20 ± 0.62	0.77 ± 0.09	3.15 ± 0.11	2.31 ± 0.15	1.69 ± 0.32	3.40 ± 0.52	0.41
	Lactose	10.10 ± 0.25	2.98 ± 0.11	0.96 ± 0.07	2.01 ± 0.10	_	0.30 ± 0.02	1.01 ± 0.46	0.30
	Fructose	49.51 ± 0.55	18.36 ± 0.32	1.01 ± 0.10	2.20 ± 0.09	2.81 ± 0.11	1.87 ± 0.14	2.96 ± 0.26	0.37
	Galactose	39.49 ± 0.23	11.88 ± 0.12	0.19 ± 0.06	1.98 ± 0.12	1.56 ± 0.14	1.29 ± 0.21	3.27 ± 0.43	0.30
	Maltose	40.28 ± 0.54	14.57 ± 0.24	4.68 ± 0.08	2.50 ± 0.14	1.91 ± 0.12	3.26 ± 0.33	3.74 ± 0.44	0.36

The 2,3-BD production of the Δ*adhE* and Δ*pta* mutants increased similarly with galactose, fructose, and maltose (Table 3). These carbon sources are consumed in the glycolytic pathway, similar to glucose. The highest production of 2,3-BD was observed in Δ*adhE*, but the Δ*pta* strain exhibited high acetoin production.

The wild-type KG1 was ineffective in converting lactose to 2,3-BD because of its low lactose consumption rate. Galactose consumption slightly increased in the mutants, but the reason remains unclear. The 2,3-BD production from lactose decreased in mutants. However, the consumption and 2,3-BD production from galactose in mutants were better than those in the parent strain KG1. Overall, the mutants enhanced 2,3-BD production with glucose, galactose, fructose, or maltose, respectively, but 2,3-BD production decreased with the use of lactose.

### Metabolic profiles of Δ*adhE* Δ*ldhA* strain and production of 2,3-BD by fed-batch culture

**Figure 4:**
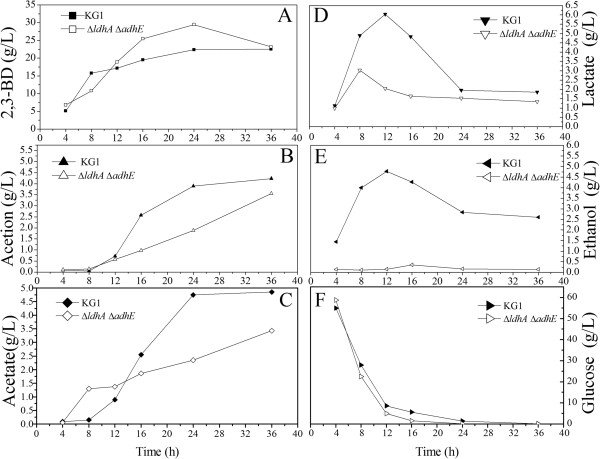
**Metabolite profiles of the *K. pneumoniae *parent strain (KG1) and *ΔldhA ΔadhE *mutant in the batch culture.** The curves were calculated from one measurement of three experiments. 2,3-BD, 2,3-Butanediol. **A)** 2,3-BD; **B)** Acetion; **C)** Acetate; **D)** Lactate; **E)** Ethanol; **F)** Glucose.

As shown in Figure 1 and Figure 2, the *ldhA* deletion could promote cell growth, and *adhE* deletion could increase 2,3-BD yield, so a double-mutant Δ*adhE* Δ*ldhA* was constructed to produce 2,3-BD with high efficiency. As shown in Figure 4, the 2,3-BD production was achieved at 24 h by the double-knockout mutant, and reached 29.48 g/L. The productions of lactate and ethanol decreased significantly and the rate of glucose consumption increased, compared with the parent strain, respectively.

**Figure 5:**
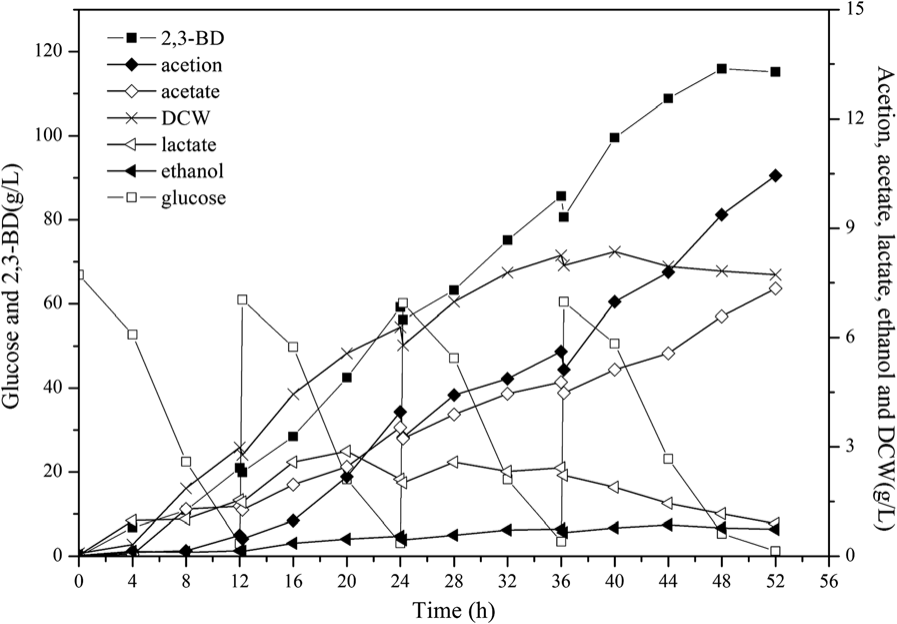
**Fed-batch fermentation profiles of Δ*ldhA* Δ*adhE* mutant.** DCW, dry cell weight; 2,3-BD, 2,3-Butanediol. The curves were calculated from one measurement of three experiments.

Fed-batch culture could enhance 2,3-BD production and reduce the cost of production by taking full advantage of the substrate. Thus, fed-batch fermentations were conducted in a 5-L bioreactor. As shown in Figure 5, a fed-batch culture was carried out by feeding glucose solution. In order to maintain optimum levels of glucose, about 750 mL of 600 g/L glucose was added to the bioreactor throughout the fermentation. After 52 h, the fed-batch fermentation of the Δ*adhE* Δ*ldhA* strain produced about 116 g/L of 2,3-BD, whereas the time courses of the other metabolites had the same tendency with the batch culture (Figure 5).

## Discussion

Numerous attempts have been made to reduce the carbon flux to by-products and increase the NADH availability for 2,3-BD through metabolic engineering in several strains [23-25]. In this study, to channel the reducing power toward the desired redox-reaction, genes encoding LDH, ADH, and PTA (*ldhA*, *adhE*, and *pta*) of *K. pneumoniae* were knocked out. First, lactate, ethanol, and acetate are the major byproducts that consume carbon flux from carbon in 2,3-BD production. Second, the formation of lactate and ethanol requires NADH, which competes with 2,3-BD production process.

Genetically engineered strains always exhibit a slow growth speed because of the changes in the metabolic pathway [17,26]. However, in our system, the mutants showed different cell growth patterns (Figure 2). The Δ*ldhA* strain showed faster growth than the Δ*pta*, Δ*adhE*, and parent strains. The main reason for the increase in cell growth may be due to the decrease in lactate production, resulting in a low acidification rate of the media. Indeed, compared to the wild-type strain, NaOH spent by the *ldhA* mutant in batch fermentation decreased by 70%. Deletion of the *ldhA* maybe increased the carbon flux to acetyl-CoA, which was subsequently converted to ethanol and biomass. However, 2,3-BD production did not improve significantly, which is consistent with a previous study [27]. The increased production of ethanol was attributed to the redirection of carbon flux and NADH to ethanol production.

In anaerobic conditions, cells are forced to produce different byproducts (mostly ethanol) for the balancing of NADH_2_ and the 2,3-BD yield is always limited. However, in our system, ethanol amounts were low (as shown in Table 2 and Table 3). We have repeated this experiment more than 10 times, and obtained the same result every time. The NADH regeneration was mainly caused by the ethanol, acetoin and succinic acid pathway [28]. But under microaerobic conditions, the other pathway was perhaps also involved, such as: NADH_2_ + ADP + 1/2 O_2_ → NAD^+^+ATP + H_2_O [29]. On the other hand, Ji *et al*. also reported that the 2,3-BD yield of the mutant with inactivation of acetaldehyde dehydrogenase gene increased by 6.67%, whereas ethanol and acetoin concentration was 0.45 g/L and 0.98 g/L, decreased by 92.21% and 64.1%, respectively [11]. The values of reducing equivalent recovery (R_H_) (Table 2) were scattered between 0.54 and 1.24, which also suggests that there are existed other NADH regeneration pathway.

The Δ*pta* and Δ*adhE* strains showed slower cell growth than the wild-type strain in the first 8 h, whereas the Δ*adhE* strain achieved the highest biomass accumulation. The inactivation of ADH and PTA genes significantly increased 2,3-BD production. The production of acetate and ethanol dramatically decreased in both mutant strains, which redirected the carbon flux to 2,3-BD production, and the increased intracellular NADH concentration and the NADH/NAD ^+^ ratio altered the metabolite spectrum of the mixed acid-2,3-BD fermentation pathway. The most direct result was the reduction in acetoin accumulation with increasing 2,3-BD production. However, the highest production of 2,3-BD was obtained by the Δ*adhE* strain compared to the mutants and wild-type strains. This result may be due to the reduction in acetoin and ethanol production in Δ*adhE*, which decreased by 62.73% and 89.65%, respectively, compared with those in the parent strain.

The effect of LDH, ADH, and PTA disruption was tested using five different carbon sources. The *ldhA* mutant showed an improvement in carbon source consumption rates, biomass production, and 2,3-BD production with all carbon sources, except for lactose, compared to the control cells. As shown in Table 2 and Table 3, the yield of ethanol was very high when maltose was used as the substrate in both mutants and wild-type strains, and 2,3-BD production was not the highest, because the ethanol formation pathway competed with 2,3-BD production pathway for the NADH and carbon flux supply. We also found that, when galactose was used as the carbon source in a 36-h flask cultivation, the lactate content was much lower in the mutants than in the wild-type strains. This is also interesting; perhaps knockout of these genes partially blocks the lactate metabolic pathway, providing more energy and sources for the strains to produce 2,3-BD. The 2,3-BD production increased significantly in the *pta* and *adhE* mutants from all carbon sources, except lactose. However, their biomass production and carbon source consumption rates decreased compared with those of the parent strain.

The carbon source consumption, biomass production, and 2,3-BD production rates decreased significantly in the mutants when lactose was used as the major carbon source. Lactose consumed only 10.1 g and 17.78 g in Δ*pta* and Δ*adhE* strains for 36 h, respectively, which was 20.44% and 35.39% of that of the parent strain. Thus, the production of byproducts significantly decreased. In *K. pneumoniae* strains, lactose hydrolysis is the limiting step because lactose must first be hydrolyzed by enzymatic processes into monosaccharides, which can then be further converted into butanediol in a second stage when lactose is used as a major carbon source [30]. The deletion of *ldhA*, *adhE*, and *pta* negatively affected the enzyme level of lactose metabolism, which caused the slow lactose utilization rate. The lactose permease activity of KG1, *ΔadhE*, *ΔldhA* and *Δpta* was 8.07 nmol/minute/g,4.91 nmol/minute/g, 4.02 nmol /minute/g,, and 3.98 nmol/minute/g, respectivity. The β-galactosidase activity of KG1, *ΔadhE*, *ΔldhA* and *Δpta* was 0.45 IU/mg, 0.28 IU/mg, 0.36 IU/mg and 0.27 IU/mg, respectively. The low activity of lactose permease and β-galactosidase maybe the reason for inefficient lactose consumption. However, the reasons for the altered lactose fermentation in mutants need further study.

## Conclusions

The engineering strategy pursued in this study, which was based on the redirection of carbons toward the production of byproducts, led to the development of *K. pneumoniae* strains with high efficiency for 2,3-BD production. In conclusion, 2,3-BD production was dramatically improved with knockout of *adhE* and *pta.* Knockout of *ldhA* could advance faster cell growth and shorter fermentation time. The double-mutant strain with deletion of *adhE* and *ldhA* resulted in accelerated fermentation and higher 2,3-BD production, although the best fermentation results in our engineered strain are almost at the same level as in wild-type microorganisms. However, we expanded the source strain for 2,3-BD fermentation and decreased the fermentation time, but we think that even genetic engineering is more efficient than traditional screening for 2,3-BD fermentation. In the near future, we should focus on optimizing the fermentation and purification to obtain a high 2,3-BD yield. In summary, these results provide new insights for industrial production of 2,3-BD.

## Materials and methods

### Plasmids, strains, and construction of plasmids

*K. pneumoniae* KG1, which was deposited at the China Center of Industries Culture Collection (CICC 10781), was used for the parent strain for 2,3-BD production [19]. The plasmids and strains used in this study are listed in Table 4. The *Escherichia coli* strain DH5α was used for the construction and amplification of plasmids. Total genomic DNA of *K. pneumoniae* and *E. coli* cells were extracted using the AxyPrep Bacterial Genomic DNA Miniprep kit. The primers used in this study are listed in Table 5.

**Table 4:** Strains and plasmids used in this work

**Strain, plasmid**	**Genotype, properties**	**Source or reference**
Strains		
*K. pneumoniae*		
KG1	parent strain (KG1)	[19]
Δ*ldhA*	A lactate-deficient mutant of KG1	This work
Δ*adhE*	An alcohol dehydrogenase-deficient mutant of KG1	This work
Δ*pta*	An phosphate acetyltransferase gene-deficient mutant of KG1	This work
Δ*ldhA* Δ*adhE*	A lactate and alcohol dehydrogenase, both deficient mutants of KG1	This work
*E. coli*		
DH5α	Φ80 *lacZ*ΔM15 Δ*lacU169 recA1 endA1 hsdR17 supE44 thi-1 gyrA relA1*	TaKaRa
S17-1 *λpir*	*TpR SmR recA thi-1 pro* hsdR-M^+^RP4: 2-Tc:Mu: Km Tn7 *λpir*	[31]
Plasmids		
pRE112	Suicide vector Cm^R^ SacB *oriT oriV*	[31]
p-*ldh*A’	Cm^R^, pRE112 derivative, where a 625-bp DNA fragment containing the *ldhA*’ was inserted	This work
p-*adh*E’	Cm^R^, pRE112 derivative, where a 872-bp DNA fragment containing the *adhE*’ was inserted	This work
p-*pta*’	Cm^R^, pRE112 derivative, where a 901-bp DNA fragment containing the *pta*’ was inserted	This work

**Table 5:** Primers used in this work

**Primer name**	**Primer sequences**
*ldh*A’-up	5′-CGG*GGTACC*ACGGTTGCGAACGGTATGTA-3′(*Kpn* I)
*ldh*A’-down	5′-C*GAGCTC*AGTGGTCTCCGAAATGCTGA-3′(*Sac* I)
*adh*E’-up	5′-CAT*GCATGC*ACCATCGTACGTAA AGGTGC-3′(*Sph* I)
*adh*E’-down	5′-C*GAGCTC*TTCGGAATACCCAGCTCAGC-3′(*Sac* I)
*pta*’ -up	5′-CAT*GCATGC*CAACTACATCAACGCCGACTGG-3′(*SphI*)
*pta*’-down	5′-C*GAGCTC*CGCTTTGTACGTGGTGTTACCG-3′(*SacI*)
*cat*-up	5′-CGGGCCCTAAATACCTGTGACGGAAGAT-3′
*cat*-down	5′-ATCGGGCCCTATCACTTATTCAGGCGTAGC-3′
A-down	5′-TACAAAACCAGCACCGTCCCT-3′
P-down	5′-CGCTGTGGATGACCCGCAACG-3′
L-down	5′-CCGTCCGACACTTTACCTTCC-3′

The *ldhA*’ fragment (an 805 bp segment of truncated *ldhA* gene) (GeneID: 5339517 [Genbank]) was amplified by PCR using total genomic DNA as a template and primers *ldh*A’-up and *ldh*’A-down, which were designed using the sequence information of *ldhA* of *K. pneumoniae*. The PCR mixture consisted of 1 ng of genomic DNA, 0.2 mmol each dNTP, 0.2 μmol each primer, 2 μL of rTaq PCR buffer, and 1 unit of rTaq DNA polymerase (TaKaRa, Dalian, China) in a total volume of 20 μL. The PCR reaction was carried out at 95°C for 5 minutes, followed by 30 cycles at 95°C for 40 s, 63°C for 90 s, 72°C for 120 s, and a final extension step of 72°C for 10 minutes. The *adhE*’ (an 872-bp segment of truncated *adhE* (GeneID: 5341804 [Genbank]) gene) and *pta*’ (an 901-bp segment of truncated *pta* (GeneID: 5338823 [Genbank]) gene) fragments were amplified by PCR using the corresponding primers. The PCR reaction procedure was the same as that of *ldhA*, but the annealing temperatures were 52°C and 57°C, respectively.

The *ldhA*’ fragment was cloned into the pRE112 [31] suicide vector after digestion with *Kpn* I and *Sac* I, and then 180 bp was digested by *Sph* I, which resulted in vector p-*ldh*A’. The strain containing the p-*ldh*A’ plasmid was used as a donor in conjugation with KG1. Transconjugants were selected for both chloromycetin resistance from pRE112 insertion and ampicillin resistance because the KG1 strain was resistant to ampicillin, whereas *E. coli* S17-1 was sensitive. To construct p-*ad*hE’, the DNA fragment of *adhE*’ was cleaved by *Sph* I and *Sac* I, and then ligated into the *Sph* I and *Sac* I sites of pRE112. The p-*pta*’ plasmid was constructed by cloning the *pta*’ DNA fragment into the vector pRE112 using the restrictions sites *SphI* and *SacI*.

### Construction of the gene-deficient mutants

To construct the gene-deficient mutants, *E. coli* S17-1, containing the plasmids p-*ldh*A’, p-*adh*E’, and p-*pta*’, was used as a donor in conjugation with KG1. Transconjugants were selected by Luria-Bertani (LB) plates with 100 μg/mL ampicillin and 30 μg/mL chloromycetin. PCR was used to confirm that the transconjugants were the correct insertion mutants. Oligonucleotides primer *cat*-up and primer *cat*-down were designed to amplify a 940-bp segment of p-*ldh*A. Total DNA from KG1 and the four transconjugants were used as a template, and the p-*ldh*A plasmid DNA was used as a positive contrast. Primer *cat*-up and primer L-down were used to verify the gene deletion of fixed-point validation. The *ldhA* mutant strains were verified by PCR using the primers cat-up and L-down, with the 5′ region of the *cat* (5′-CGGGCCCTAAATACCTGTGACGGAAGAT-3′) and the 3′ region of the chromosome of *K. pneumoniae* (5′-CCGTCCGACACTTTACCTTCC-3′). The *adhE* mutant strains were also verified by PCR using primers *cat*-up and A-down, whereas the *pta* mutant strains were verified by PCR using primers *cat*-up and P-down.

To make the Δ*adhE* Δ*ldhA* double mutant, the Δ*ldhA* was cultured on 10% sucrose plates to select double-crossover transconjugants. Proper in-frame deletion of the *ldhA* gene was verified by PCR. And *E. coli* S17-1, containing the plasmids p-*adh*E’ as described above was used as a donor in conjugation with *K. pneumoniae* Δ*ldhA*. Correct integration of the *adhE* deletion was confirmed by PCR using primers as described above.

### Media and cultivation conditions

The *E. coli* strain DH5α was incubated in LB medium with 30 μg/mL kanamycin or 25 μg/mL chloramphenicol resistances for plasmid maintenance. The glucose fermentation medium (pH 7.0) was composed of glucose (80 g/L), yeast extract (10 g/L), KH_2_PO_4_ (10 g/L), K_2_HPO_4_ (7.2 g/L), (NH_4_)_2_SO_4_ (2 g/L), sodium citrate (4 g/L), and trace element solution (1 mL). *K. pneumoniae* was cultured in the glucose fermentation medium at 35°C and 150 rpm.

The trace element solution was composed of the following: 3 g/L ethylenediaminetetraacetic acid (EDTA); 0.09 g/L CaCl_2_ ∙ 2H_2_O; 0.90 g/L ZnSO_4_ ∙ 7H_2_O; 0.60 g/L FeSO_4_ ∙ 7H_2_O; 200 mg/L H_3_BO_3_; 156 mg/L MgCl_2_ ∙ 2H_2_O; 80 mg/L Na_2_MoO_4_ ∙ 2H_2_O; 60 mg/L CoCl_2_ ∙ 2H_2_O; 60 mg/L CuSO_4_ ∙ 5H_2_O; and 20 mg/L KI. The pH of the trace element solution was adjusted to 4.00 with NaOH, and then the solution was autoclaved.

Seed culture (10%, v/v) was inoculated into the fermentation medium and batch and fed-batch fermentation was carried out in a 5-L stirring bioreactor (Biostat A plus, B Braun, Melsungen, Germany) with a working volume of 3 L. The fermentation was performed at 37°C with the aeration rate of 1.0 volume per volume per minute (vvm) and agitation speed was automatically changed to maintain 15% dissolved oxygen (DO) level, respectively. When the pH decreased to 6.5, it was controlled at 6.5 automatically by adding 3 M NaOH.

### Analytical methods

The protein concentration in the extracts was measured according to the Bradford method using the Bio-Rad protein reagent and ovalbumin as the standard [32]. Biomass was determined using a UV-visible spectroscopy system (8453, Agilent, Palo Alto, CA., USA) at 600 nm with appropriate dilution, and was converted to the dry cell weight (DCW). The intracellular NADH and NAD^+^ concentrations were measured by procedures presented by Ji *et al*. [17]. The concentrations of glucose, 2,3-BD, acetic acid, lactic acid, acetoin, alcohol, and acetoin were determined by high-performance liquid chromatography using an Aminex HPX-87H column at 65°C, with 5 mM H_2_SO_4_ as the mobile phase at a flow rate of 0.6 mL/minute.

### Enzyme activity measurements

ADH activity was determined according to the method described by Cunningham *et al*. [33]. To determine the enzyme activity, cells were harvested after 18 h of culture under anaerobic conditions, washed with MOPS buffer (50 mM, pH 7.4, containing 4 mM DTT, 10 mM MgSO_4_, and 10 μM MnSO_4_), and resuspended with MOPS buffer up to 2.5 mL. The cells were then processed by ultrasonication to collect the crude extracts. The ADH reaction mixtures contained the following in a total volume of 1 mL: 75 nmol NAD^+^, 100 nmol CoA, 10 μL of 1.0 M acetaldehyde, 20 μL of crude extracts, and CHES buffer (12 mM, pH 8.5). The ADH reaction mixture contained the following in a total volume of 1 mL: 75 nmol NAD^+^, 20 μL of alcohol, 20 μL of crude extracts, and sodium pyrophosphate solution (12 mM, pH 8.5). One unit of enzyme activity was defined as the generation of 1 μmol NADH per minute.

The LDH activity was determined according to the method established by Tarmy *et al*. [34]. The enzyme reaction mixtures contained the following in a total volume of 1 mL: 0.33 mM NADH, 30 mM sodium pyruvate, 20 μL of crude extracts, and potassium phosphate buffer. The cells were harvested after 12 h of culture under aerobic conditions. The crude extracts were extracted using the same method previously mentioned, except for the addition of potassium phosphate buffer instead of MOPS buffer. The unit of enzyme activity was defined as the oxidation of 1 μmol NADH per minute.

The PTA activity was determined according to previous studies, with minor modifications [35]. The enzyme reaction mixture contained the following in a total volume of 3.005 mL: 2.95 mL of 0.1 M potassium phosphate buffer (pH 7.4), 20 μL of 0.2 mM acetyl-CoA, 30 μL of 0.08 mM DTNB, and 5 μL of dialyzed extract. The cells were harvested after 18 h of culture under aerobic conditions. The crude extracts were extracted using the same method previously mentioned, except for the addition of potassium phosphate buffer instead of MOPS buffer. One unit of PTA was defined as the amount of enzyme that generates 1 nmol CoA per minute.

The activity of β-galactosidase was determined by our previous reported [36]. The lactose permease activity was determined as the method of maltose permease activities [37], with minor modifications (lactose was used as the maltose). One unit of lactose permease was defined as the amount of lactose uptake by 1 g DCW biomass per minute.

The method for calculating the NADH_2_ balance and carbon recovery was as previously reported [38]. The biochemical reactions involved in the metabolism of glucose by *K. pneumoniae* under microaerobic conditions can be written as follows [4,29,38]:

Glucose → 2acetic acid + 2NADH + 4ATP + 2CO_2_ + 2fomate-2H_2_O

Glucose → 2ethanol + 2ATP + 2CO_2_ + 2formate + 2H_2_O-2 NADH

Glucose → 2,3-BD + 2ATP + 2CO_2_ + NADH + 2H_2_O

Glucose → Aceion + 2ATP + 2CO_2_ + 2NADH + 2H_2_O

Glucose → 2lactic acid + 2ATP + 2H_2_O

Glucose → 2succinic acid + 2ATP + 2H_2_O-2 NADH

The NADH2 balance and carbon recovery can be calculated as follows:

$$\begin{array}{l} R_{H}=\left(2\;q_{\text{EtOH}}+2\;q_{\text{SUC}}\right)/(2\;q_{\text{HAC}}+q_{\text{BD}}+2\;q_{\text{ACE}}\\ \;+13.2\mu )\\ R_{C}=(2\;q_{\text{EtOH}}+2\;q_{\text{SUC}}+2\;q_{\text{HAC}}+q_{\text{BD}}+2\;q_{\text{ACE}}\\ \;+13.2\mu +2\;q_{\text{Lac}})/q_{s} \end{array}$$

Where q_ACE_ = specific formation rate of aceion mmol/g/h; q_BD_ = specific formation rate of 2,3-butanediol mmol/g/h; q_EtOH_ = specific formation rate of ethanol mmol/g/h; q_HAc_ = specific formation rate of acetic acid mmol/g/h; q_Lac_ = specific formation rate of lactic acid mmol/g/h; q_S_ = specific consumption rate of glucose mmol/g/h; q_SUC_ = specific formation rate of succinic acid mmol/g/h; R_C_ = carbon recovery; R_H_ = reducing equivalents recovery; μ = specific growth rate/h.

## Abbreviations

2,3-BD: 2,3-Butanediol; ADH: acetaldehyde dehydrogenase; bp: base pairs; DCW: dry cell weight; DO: dissolved oxygen; KG1: *K. pneumonia* parent strain; LB: Luria-Bertani; LDH: lactate dehydrogenase; NAD+: nicotinamide adenine dinucleotide; NADH: nicotinamide adenine dinucleotide plus hydrogen; PCR: polymerase chain reaction; PTA: phosphotransacetylase; qACE: specific formation rate of aceion mmol/g/h; qBD: specific formation rate of 2,3-butanediol mmol/g/h; qEtOH: specific formation rate of ethanol mmol/g/h; qHAc: specific formation rate of acetic acid mmol/g/h; qLac: specific formation rate of lactic acid mmol/g/h; qS: specific consumption rate of glucose mmol/g/h; qSUC: specific formation rate of succinic acid mmol/g/h; RC: carbon recovery; RH: reducing equivalents recovery; μ: specific growth rate/h.

## Competing interests

The authors declare that they have no competing interests.

## Authors’ contributions

XG designed and performed the experiments, and drafted the manuscript; CC, YW, CL and MW performed some experiments, analyzed data and drafted the manuscript; CZ and YC provided some reagents, helped to design the experiment and drafted the manuscript; HP and DX designed the experiments and supervised the project; XG, HP and DX analyzed the data and critically revised the manuscript. All of the authors read and approved the manuscript.
